# Comparable bidirectional neutrophil immune dysregulation between Kawasaki disease and severe COVID-19

**DOI:** 10.3389/fimmu.2022.995886

**Published:** 2022-09-08

**Authors:** Kuang-Den Chen, Ying-Hsien Huang, Wei-Sheng Wu, Ling-Sai Chang, Chiao-Lun Chu, Ho-Chang Kuo

**Affiliations:** ^1^ Kawasaki Disease Center, Kaohsiung Chang Gung Memorial Hospital, Kaohsiung, Taiwan; ^2^ Department of Pediatrics, Kaohsiung Chang Gung Memorial Hospital, Kaohsiung, Taiwan; ^3^ nstitute for Translational Research in Biomedicine, Kaohsiung Chang Gung Memorial Hospital, Kaohsiung, Taiwan; ^4^ College of Medicine, Chang Gung University, Taoyuan, Taiwan; ^5^ Department of Electrical Engineering, National Cheng Kung University, Tainan, Taiwan; ^6^ Department of Respiratory Therapy, Kaohsiung Chang Gung Memorial Hospital, Kaohsiung, Taiwan

**Keywords:** kawasaki disease, COVID-19, aged neutrophils, overactivation, single-cell RNA sequencing

## Abstract

Kawasaki disease (KD), a multisystem inflammatory syndrome that occurs in children, and severe acute respiratory syndrome coronavirus 2 (SARS-CoV-2 or COVID-19) may share some overlapping mechanisms. The purpose of this study was to analyze the differences in single-cell RNA sequencing between KD and COVID-19. We performed single-cell RNA sequencing in KD patients (within 24 hours before IVIG treatment) and age-matched fever controls. The single-cell RNA sequencing data of COVID-19, influenza, and health controls were downloaded from the Sequence Read Archive (GSE149689/PRJNA629752). In total, 22 single-cell RNA sequencing data with 102,355 nuclei were enrolled in this study. After performing hierarchical and functional clustering analyses, two enriched gene clusters demonstrated similar patterns in severe COVID-19 and KD, heightened neutrophil activation, and decreased MHC class II expression. Furthermore, comparable dysregulation of neutrophilic granulopoiesis representing two pronounced hyperinflammatory states was demonstrated, which play a critical role in the overactivated and defective aging program of granulocytes, in patients with KD as well as those with severe COVID-19. In conclusion, both neutrophil activation and MHC class II reduction play a crucial role and thus may provide potential treatment targets for KD and severe COVID-19.

## Introduction

Kawasaki disease (KD) was first reported with an unknown etiology more than 50 years ago by Dr. Kawasaki in Japan. The five major clinical diagnosis criteria of KD (1) include oral mucosa changes (fissure lips, strawberry tongue, and oral mucosal inflammation), bilateral conjunctivitis, enlarged neck lymph nodes, limb induration (subsequent desquamation), and polymorphic skin rash (2). However, the detailed mechanisms underlying the pathogenesis of KD is complex and remains inconclusive (3).

Recent outbreaks of the SARS-CoV-2 pandemic in 2019-2021 (COVID-19) have been associated with a sharp increase in the incidence of multisystemic inflammatory syndrome in children (MIS-C), suggesting a common etiology and pathophysiology with KD (4–6). Certain signs or symptoms are specific to KD and go beyond the five major symptoms, such as induration over the bacillus Calmette-Guérin (BCG) vaccination site and coronary arteritis, dilatation, or aneurysm formation. Coronary artery involvement has also been found in rheumatologic or infectious diseases but are primarily (more than 95%) found in KD patients, MIS-C patients and now also experience of COVID-19 vaccine-related adverse events among adolescents and youth (7–9). Meanwhile, BCG vaccination has also been reported to have some protective role in COVID-19 (10, 11). BCG induration in patients with KD was considered a T cell immune response, much like the type 4 hypersensitivity of the tuberculin skin test (PPD skin test), while T cell response has also been reported in COVID-19 (12, 13).

Hypercytokinemia, or “cytokine storm,” refers to a set of clinical conditions caused by excessive immune reactions and has been recognized as a leading cause of both Kawasaki disease (KD) (14) and severe COVID-19 (15, 16). A recent study profiled MIS-C, adult COVID-19, and healthy individuals using single-cell RNA sequencing and found elevated alarmins and decreased antigen presentation signatures, thus indicating myeloid dysfunction in both MIS-C and COVID-19 patients (17). Currently, a study revealed that MIS-C and KD shared same fundamental nature of the host immune response continuum as COVID-19 which was found to be predominantly IL15/IL15RA-centric cytokine storm (18). Thus, we determined to characterize the cytokine responses in COVID-19, focusing on the impact of disease severity by comparing those in KD, as such may provide important clues regarding the underlying pathogenesis of both diseases. Monocyte/macrophage-derived interleukin (IL)-1β and epithelial cell-derived IL-6 were unique features of SARS-CoV-2 infection compared to the viral and bacterial causes of pneumonia. Despite having no evidence of active infection, MIS-C patients displayed elevated S100A-family alarmins and decreased antigen presentation signatures, which indicated myeloid dysfunction (17). Therefore, COVID-19 and KD may share some innate inflammatory or immune responses and thus need to be analyzed together. In this study, we aimed to analyze the shared innate immune characteristics between COVID-19 and KD using single-cell RNA sequencing profiling. To unravel the complexity of immune response in COVID-19 and KD, we performed a detailed immune cell phenotyping and transcriptomics analyzed at the single-cell level on whole blood cells. We compared data from children with KD during acute phase and patients with SARS-CoV-2 acute infection, and then analyzed the hyperinflammatory cell types and their associated molecular signatures.

## Results

### Comparing single-cell transcriptomes of peripheral immune cells between Kawasaki disease and COVID-19 patients

To characterize the immunological properties of patients with Kawasaki disease, we performed droplet-based single-cell transcriptomic profiling of whole blood cell (WBC) specimens from three patients (KD) and two febrile controls (FC) at Kaohsiung Chang Gung Memorial Hospital. Furthermore, we obtained the COVID-19 datasets were obtained from the Sequence Read Archive (SRA) public repository (five data sets for severe influenza (FLU), nine datasets for COVID-19 (four for mild COVID-19, five for severe COVID-19), and four datasets for healthy controls (HC) in BioProject PRJNA629752 according to GSE149689 in the GEO database).

**Figure 1:**
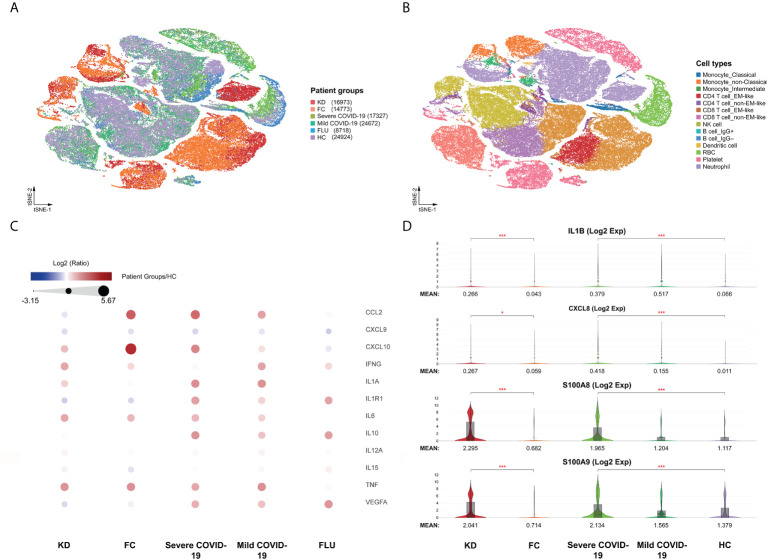
Immunological characterization of blood cells from Kawasaki disease and COVID-19 patients. **(A)** t-SNE projection of the blood cells from patients with Kawasaki disease (KD) (three samples), febrile controls (FC) (two samples), patients with COVID-19 (four samples for mild COVID-19, five samples for severe COVID-19), and healthy controls (HC) (four samples). Each dot corresponds to a single cell, colored according to group information. **(B)** t-SNE projection colored by cell types. Canonical cell markers were used to label clusters by cell identity as represented in the t-SNE plots of **Figure S1**. **(C)** Direct comparison of known cytokines among single-cell datasets that may be involved in hypercytokinemia of both KD and severe COVID-19 diseases. **(D)** Violin plots showing the comparable expression of canonical hypercytokinemia markers IL1B, CXCL8, S100A8, and S100A9 under different conditions. Genes were considered differentially expressed according to the *P* values from the Mann-Whitney *U* test with a false discovery rate (FDR) < 0.05 (*), or < 0.0005 (***).

After completing the unified single-cell analysis procedure with stringent quality control (see Methods), we obtained approximately 625 million unique transcripts from 107,387 nuclei from the immune cells of all samples. Among these cells, 16,973 cells (15.8%) came from KD patients, 14,773 cells (13.8%) came from FC, 17,327 cells (16.1%) came from severe COVID-19, 24,672 cells (23%) came from mild COVID-19, 8,718 cells (8.1%) came from FLU, and 24,924 cells (23.2%) came from HC subjects. We performed integrative analysis to harmonize all 23 datasets, followed by graph-based clustering and non-linear dimensionality reduction using t-Distributed Stochastic Neighbor Embedding (t-SNE) in order to visualize communities of similar cells by reducing dimensionality based on highly variable genes using the Loupe^®^ browser (**Figure 1A**). We resolved 14 distinct cell types (**Figure 1B**) assigned from 45 t-SNE-identified different clusters, unbiased by datasets from all the experimental batches of scRNA-seq studies based on well-known marker genes (**Figure S1**).

**Figure 2:**
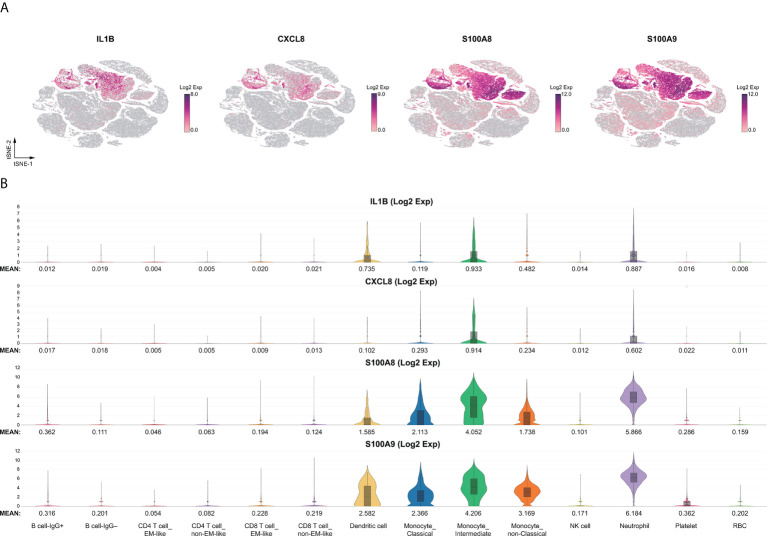
Immune landscape of the comparable myeloid inflammatory markers. The comparable innate inflammatory response between KD and severe COVID-19 with elevated myeloid IL1B, CXCL8, and alarmins of the S100A family were heightened in neutrophils. **(A)** t-SNE projection of representative gene expression patterns for IL1B, CXCL8, S100A8, and S100A9. **(B)** Violin plots of normalized log2 expression of the selected activation genes across the 14 different cell types.

We performed the first direct comparison of cytokine profiles in both diseases. Genes were considered differentially expressed when the false discovery rate (FDR) ≤ 0.05. Seven out of the 16 known cytokine markers (including IL1A, IL1R1, CCL2, IL6, IL10, CXCL10, and VEGFA) were less pronounced in KD than in severe COVID-19 patients compared to FC and HC, respectively (**Figure 1C**). Levels of the inflammasome cytokine IL1B, the neutrophil chemotaxis factor CXCL8, and alarmins S100A8 and S100A9 were comparably elevated in KD and severe COVID-19 compared to FC and HC, respectively (**Figure 1D**). However, IL15, CXCL9 and IL12A levels were not significantly altered in KD or severe COVID-19 compared to FC or HC, respectively. TNF levels did not differ between KD and its febrile controls. Furthermore, IFN-γ (IFNG) levels were more than three-fold higher in KD compared to severe COVID-19, a key observation that differentiates KD hypercytokinemia from the cytokine storm in severe COVID-19. Nevertheless, we found markedly lower levels of IFNG-induced chemokine CXCL10 in KD and CXCL9 and IL15 in both KD and severe COVID-19, suggesting blunted type II interferon signaling in both KD and severe COVID-19 conditions. Therefore, we linked comparably elevated IL1B and CXCL8 values to the emerging role of neutrophil activation in both KD and severe COVID-19. Interestingly, the four highly increased markers of IL1B, CXCL8, S100A8, and A100A9 were mainly expressed by neutrophils (**Figure 2**), so we specifically focused on neutrophils in downstream analysis.

### Features of neutrophil subsets in both KD and severe COVID-19 patients

We compared the expression patterns of the KD or severe COVID-19 condition with that of the FC or HC condition, respectively, in 15,428 neutrophils. Genes were considered differentially expressed when FDR ≤ 0.05. In the KD patient group, 474 genes were differentially expressed when compared with the FC group, while 2,265 differentially expressed genes (DEGs) appeared in the severe COVID-19 group when compared with the HC group. **Figure S2** shows 219 overlapped DEGs between the KD group and the severe COVID-19 group. Next, we aimed to identify relevant biological functions in clustered genes in terms of the ontological categorization. We found that the overlapping DEGs were highly associated with immune response, MHC class II protein complex, T cell co-stimulation, interferon-γ-mediated signaling pathway, inflammatory response, and neutrophil chemotaxis as the top-ranked biological and cellular functions (**Table 1**). Interestingly, the three functional categories of MHC class II protein complex, T cell co-simulation, and interferon-γ-mediated signaling pathway share nine MHC class II genes, while the two functional categories of inflammatory response and neutrophil chemotaxis share 11 genes involved in neutrophil activation (**Figure S3**).

**Table 1:** DAVID enrichment analysis for 219 overlapped DEGs between KD and severe COVID-19.

GOID	Term	Count	%	Genes	Fold Enrichment	FDR
GO:0006955	Immune response	32	15.61	IFITM3, IFITM2, CXCL8, CCL4L2, CXCL3, THBS1, CXCL2, CRIP1, HLA-DMA, HLA-DMB, NFIL3, IGKC, CCL4, CCL3, FCGR1A, HLA-DQA2, HLA-DQA1, HLA-DPA1, CD74, HLA-DRB5, IL1R2, OSM, PPBP, CD4, IL6, IGLV1-51, IL1B, HLA-DPB1, HLA-DRA, HLA-DRB1, PF4, HLA-DQB1	6.718	8.79E-14
GO:0042613	MHC class II protein complex	11	5.366	CD74, HLA-DMA, HLA-DRB5, HLA-DMB, HLA-DPB1, HLA-DRA, HLA-DQA2, HLA-DRB1, HLA-DQA1, HLA-DPA1, HLA-DQB1	46.97	1.54E-12
GO:0060333	Interferon-γ-mediated signaling pathway	14	6.341	HLA-DRB5, IFNGR1, STAT1, IFI30, MT2A, HLA-DPB1, HLA-DRA, FCGR1A, HLA-DMA, HLA-DMB, HLA-DQA1, HLA-DRB1, HLA-DPA1, HLA-DQB1	16.18	9.79E-09
GO:0006954	Inflammatory response	23	11.22	CSF1R, CXCL8, CCL3L1, CCL4L2, CXCR4, TNFAIP3, PPBP, FOS, CXCL3, PTGS2, CXCL2, THBS1, IL6, IL1B, NFKBIZ, CCL4, CCL3, S100A12, NLRP3, NFKBID, S100A9, S100A8, PF4	5.363	8.84E-08
GO:0030593	Neutrophil chemotaxis	11	5.366	CXCL8, CCL3L1, IL1B, CCL4L2, CCL4, CCL3, S100A12, PPBP, CXCL3, S100A9, S100A8	14.73	7.37E-07
GO:0019882	Antigen processing and presentation	10	4.878	CD74, HLA-DRB5, HLA-DMB, HLA-DPB1, HLA-DRA, HLA-DQA2, HLA-DRB1, HLA-DQA1, HLA-DPA1, HLA-DQB1	16.07	1.71E-06
GO:0031295	T cell co-stimulation	12	5.366	HLA-DRB5, CD4, LGALS1, HLA-DPB1, HLA-DRA, MAP3K8, HLA-DMA, HLA-DMB, HLA-DRB1, HLA-DQA1, HLA-DPA1, HLA-DQB1	12.46	2.61E-06
GO:0070098	Chemokine-mediated signaling pathway	10	4.878	CXCL8, CCL3L1, CCL4L2, CCL4, CCL3, CXCR4, PPBP, CXCL3, CXCL2, PF4	12.45	1.06E-05

**Figure 3:**
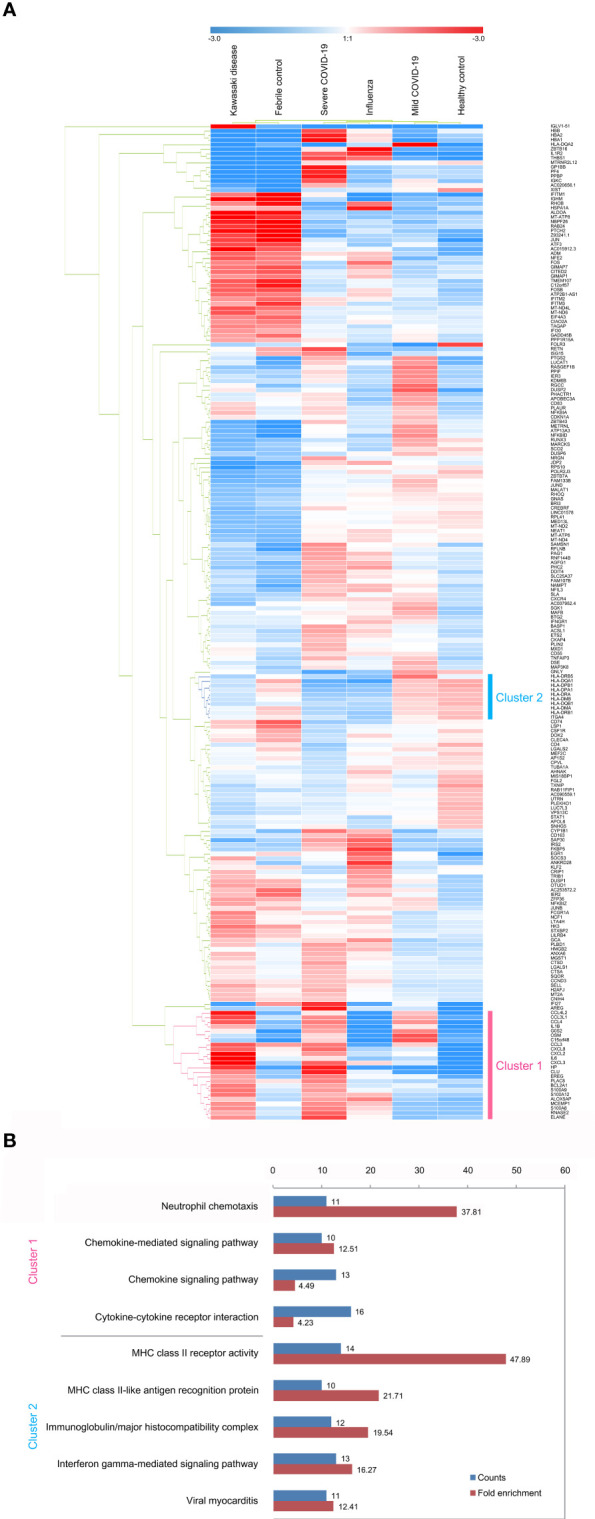
Subpopulation analysis of neutrophils. **(A)** Heatmap of 219 differentially expressed genes in neutrophil subsets across disease conditions (KD, FC, severe COVID-19, mild COVID-19, FLU, and HC). The color bars indicate gene expression clustered by hierarchical clustering for normalized gene expression levels. Cluster 1 genes (pink) represent heightened levels, whereas cluster 2 genes (cyan) are drastically reduced in both KD and severe COVID-19 compared to the controls (FC and HC, respectively). **(B)** The lists of over-represented gene ontology function of the two closely clustered genes featured by the DAVID annotation tool. The fold enrichment is defined as the ratio of the two proportions, where one is the proportion of the input 219 DEGs belonging to a certain GO term, and the other is the proportion of genes in the universal background belonging to that specific GO term. Adjusted *p* values are calculated from modified Fisher’s exact test with FDR multiple-testing correction.

Furthermore, the hierarchical cluster analysis results of the 219 DEGs represented two clusters with comparably up-regulated and down-regulated genes in both KD and severe COVID-19 among all group conditions (**Figure 3A**). The over-represented function of the two closely clustered genes between KD and severe COVID-19 featured by the DAVID annotation tool is shown in **Figure 3B**. The featured functions included neutrophil chemotaxis, chemokine signal pathway, chemokine-mediated signal pathway and cytokine-cytokine receptor interaction for the cluster 1 genes, while MHC Class II activity, immunoglobulin/major histocompatibility complex, MHC Class II-like antigen recognition protein, interferon-γ-mediated signal pathway, and viral myocarditis represented the cluster 2 genes.

**Figure 4:**
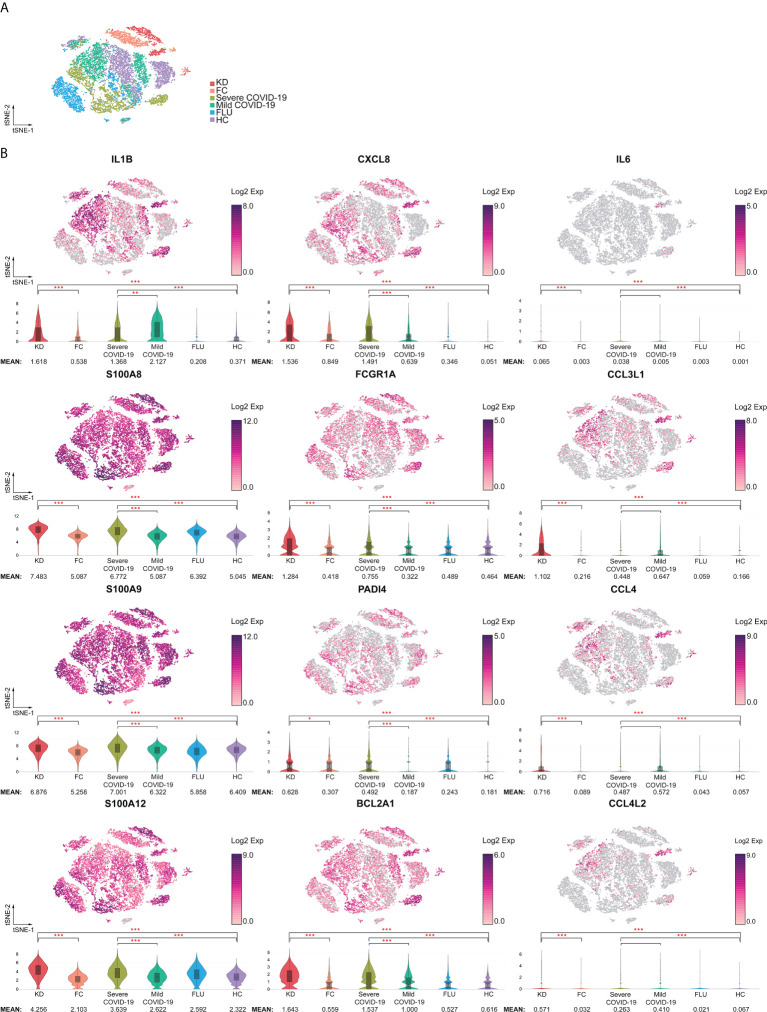
Increased activation signatures in neutrophils from both KD and severe COVID-19 patients. **(A)** t-SNE projection of neutrophils across disease conditions. **(B)** t-SNE and violin plots of normalized log2 expression levels of activation genes in cluster 1 identified in **Figure 3A**. The panel of genes was chosen based on their known role in neutrophil activation (IL1B, IL6, CXCL8, S100A8, S100A9, and S100A12), infiltration (CCL3L1, CCL4, CCL4L2, and FCGR1A), survival (BCL2A1), and neutrophil extracellular trap formation (PADI4). < 0.05 (*), < 0.005 (**) or < 0.0005 (***).

**Figure 5:**
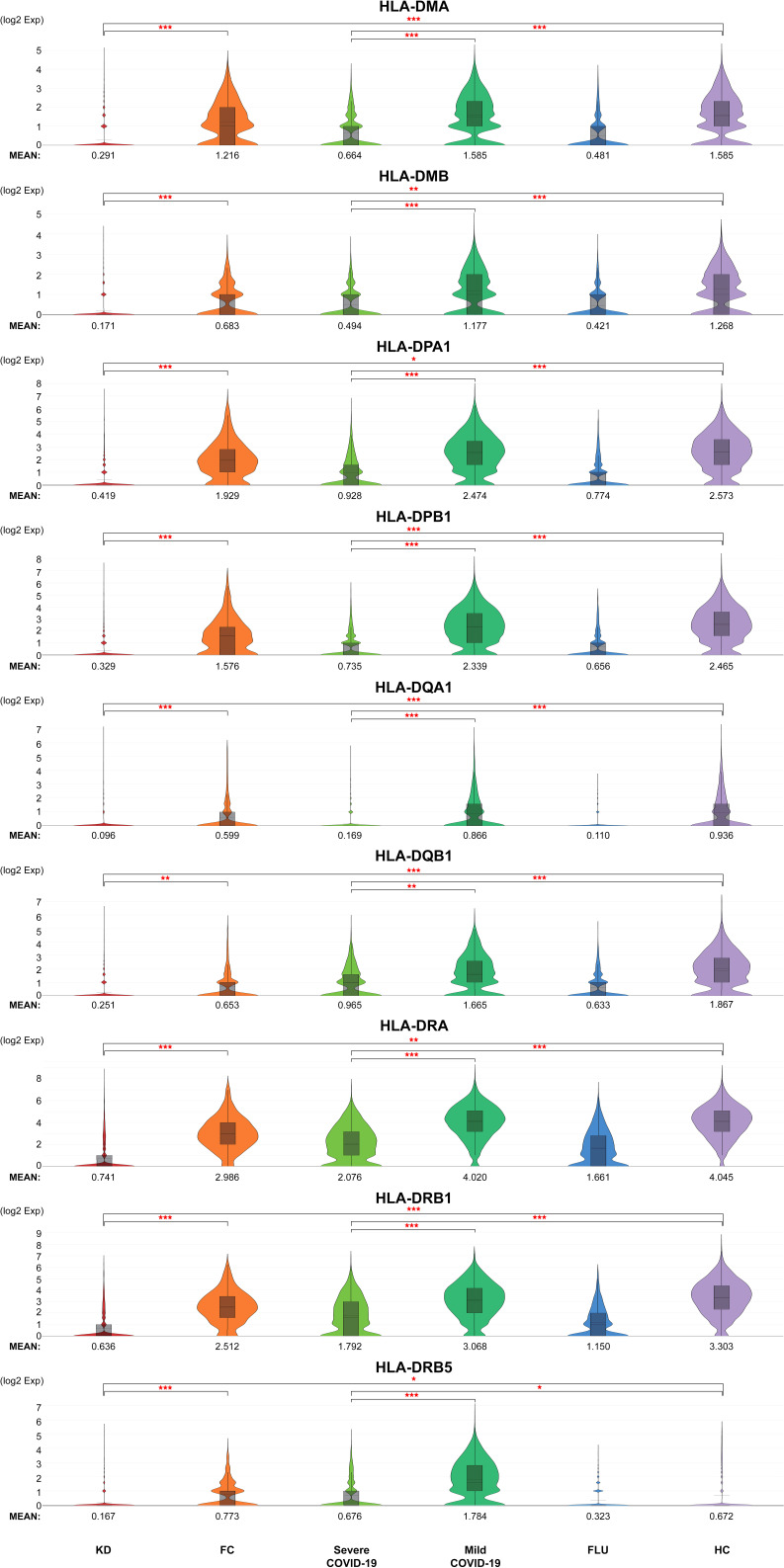
Generally reduced MHC class II in neutrophils of both KD and severe COVID-19 patients. Violin plots of normalized log2 expression levels of MHC class genes in cluster 2 identified in **Figure 3A**. The panel of genes was chosen based on their known role in antigen presentation and interferon-γ-mediated signaling pathway. < 0.05 (*), < 0.005 (**) or < 0.0005 (***).

The 12 neutrophil activation markers demonstrated a significant increase in KD when compared with FC, including IL1B, CXCL8, IL6, S100A8, S100A9, S100A12, FCGR1A, CCL3L1, PADI4, CCL4, BCL2A1 and CCL4L2 (all p <0.001, except PADI4, p <0.05) (**Figure 4**). Meanwhile, CXCL8, S100A8, S100A9, S100A12, FCGR1A, PADI4, and BCL2A1 showed significant increases in severe COVID-19 when compared with mild COVID-19 (p <0.05). All 12 markers of neutrophil activation were significantly higher in KD and severe COVID-19 when compared with adult HC (p <0.001). The eight MHC class II gene expressions were significantly decreased in the KD group compared to FC, including HLA-DMA, HLA-DMB, HLA-DPA1, HLA-DPB1, HLA-DQA1, HLA-DRA, HLA-DRB1, and HLA-DRB5, as well as in severe COVID-19 compared to mild COVID-19 (**Figure 5**). All nine MHC class II genes showed significant decreases in both KD and severe COVID-19 conditions compared to their respective control groups.

### Neutrophil maturation and aging trajectories

**Figure 6:**
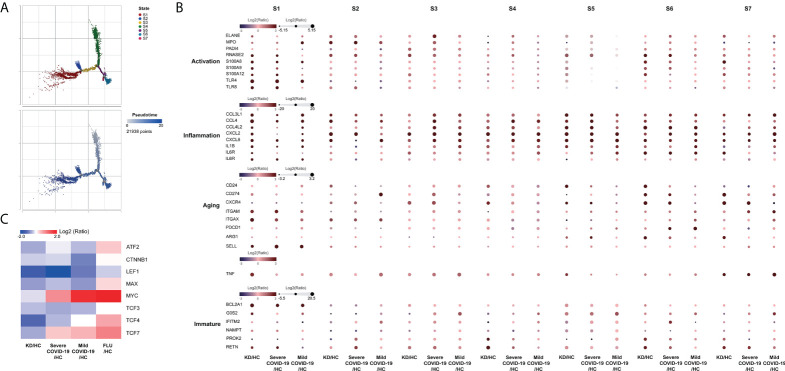
Monocle trajectory of neutrophils. **(A)** Trajectory analysis of neutrophils using Monocle 2 was colored by states (upper panel) and pseudotime maps (lower panel) showed changes in neutrophil differentiation. Cell states were inferred from the expression genes in neutrophils. **(B)** Activation-, inflammation-, aging- and immaturation-related genes in neutrophil subpopulations of different states. **(C)** Heatmap showing the significant differential expression of aging-related cell proliferation genes in neutrophils.

According to known gene signatures, we further conducted differentiating neutrophil populations along possible granulopoiesis trajectories in pseudo-time using Monocle 2 algorithm. As shown in **Figure 6A**, neutrophil maturation and aging organized on a tightly associated trajectory, starting from S4 cells and S2 cells, and ending at the left-branched S1 cells and right-branched aging neutrophils (S5-S7 cells). Neutrophil differentiation in S3 concluded with a less mature state that highly expressed CXCL2 but lower CCL4 and CXCL8 than S1, all of which are vital for neutrophil inflammation and mobilization (**Figure 6B**). S1 cells exhibited the highest expressions of alarmins S100A8, S100A9, S100A12 and toll-like receptors TLR4 and TLR8, all of which are involved in neutrophil activation and were the most mature neutrophils in both KD and Severe COVID-19 patients. S5, S6, and S7 cells exhibited relatively aged states that highly expressed CXCL4 and ARG1 but lower SELL, which accounted for the majority of aged neutrophils, while S7 cells also showed the highest TNF-α expression in both KD and severe COVID-19 patients. Furthermore, S6 and S7 cells highly expressed pyroptotic genes (such as NLRC4/5, NLRP1/12 and CASP1) in both KD and severe COVID-19 patients (data not shown). Notably, the percentages of aged neutrophils were largely increased in KD as well as severe COVID-19 patients (24.3% in KD vs. 8% in FC, 44.3% in severe COVID-19 vs. 26.9% in HC, respectively). However, there was no significant change of aged neutrophils in both influenza and mild COVID-19 patients (25.8% and 23.5%, respectively). The aging tendency of neutrophils was further concluded by reducting the transcription factors LEF1, MYC, CTNNB1, MAX and ATF2, which mediates the proliferation, survival and differentiation of granulocyte progenitor cells (**Figure 6C**). These observations indicate a comparable dysregulation of neutrophilic granulopoiesis, the occurrence of which may play a critical role in the defective aging program of granulocyte progenitors in patients with KD as well as those with severe COVID-19.

Altogether, these results suggested that the transcriptomic change for elevated neutrophil activation, inflammation and suppressive interferon-γ-mediated signaling pathway through general MHC class II reduction could be a “comparable” immune response between KD and severe COVID-19 patients compared to their controls. Overactivated and dysregulated aging neutrophils represent two pronounced hyperinflammatory states in both KD and severe COVID-19 and could be mediated *via* impaired antigen presentation.

## Discussion

On clinical grounds, both KD and severe COVID-19 have been shown to be caused by accompanying hyperinflammatory states (19, 20). In particular, inflammatory cytokines secreted by over-activated classical monocytes and macrophages have been given a central role in not only the acute phase of KD but also the severe progression of COVID-19 (21, 22). Recently, one study demonstrated that the TNF/IL1B–driven inflammatory response was dominant in COVID-19 across all types of cells among PBMCs using single-cell RNA sequencing (23). Classical monocytes in severe COVID-19 were found to be accompanied by the IFN-I response that was characterized by the up-regulation of various interferon-stimulating genes (ISGs), including ISG15, IFITM1/2/3, ISG20, IFI27, and MX1 when compared to mild COVID-19. Furthermore, an important objective in the study of KD has been identifying the pro-inflammatory cytokines that play a role in the pathogenesis of cardiac inflammation, and both TNF and IL-1 have been identified as potential *in vitro* and *in vivo* candidates. In the current study, we performed direct comparisons of cytokine expressions in KD and severe COVID and found that the expressions of IL1A, IL1R1, and TNF were about 2.6- to 10-fold higher in severe COVID-19 than in KD. Only levels of IL1B, neutrophil chemotactic CXCL8, and alarmins S100A8/S100A9 were comparably elevated in KD and COVID-19 compared to their respective controls. These four inflammatory markers were mainly expressed by neutrophils, which have a high expression of the CXCL8 receptors CXCR1 and CXCR2, which showed a significant increase in both KD and severe COVID-19 when compared with the respective controls (**Figure S4**). Furthermore, MIS-C represents a cytokine storm induced by SARS-CoV-2 with elevated inflammatory markers, including C-reactive protein (CRP), procalcitonin, neutrophilia, lymphopenia, and pro-inflammatory cytokine levels (i.e., IL-6, IL-10, ferritin, and D-dimers), that often meets the criteria for macrophage activation syndrome (MAS) of children and shares certain characteristics with KD (3, 24, 25). It has been recently reported that patients with MAS had higher absolute neutrophil counts which may also be associated with neutrophil activation (26, 27). Therefore, the mechanism underlying the neutrophil activation involved in the pathogenesis of KD could be, at least in part, comparable with that of COVID-19 as well as MIS-C. In animal models, IL-1 has been determined to be non-essential for the development of acute myocarditis, which is driven by TNF in the acute phase of KD, but plays an essential role in the subsequent development of coronary vasculitis (28). It has been demonstrated that IL1B triggers the activation of aortic infiltrated neutrophils and further leads to the formation of abdominal aortic aneurysms in an experimental murine model (29). Therefore, the comparable molecular mechanisms underlying neutrophil activation could be critical in both diseases and may contribute to unveiling the pathogenesis of KD.

Neutrophils have been demonstrated to have an important role in a variety of innate immune processes in KD. Mounting evidence has indicated that the regulation of toll-like receptors participates in the pathogenesis of KD (30) by stimulating neutrophil migration (31–33). More importantly, both neutrophil migration and transformation were associated with shock syndrome, a refractory response to IVIG and coronary artery lesions (CAL) in KD (34–36). Neutrophil activation has been reported to be enhanced with a marked increase in ROS, which contributes to the excessive formation of neutrophil extracellular traps (NETs), which has been suggested as being involved in the pathogenesis of KD (31, 37–39), as well as the severity of acute respiratory syndrome caused by SARS-CoV-2 and severe COVID-19 patients who have cardiovascular injuries (40–42). One recent study profiled whole blood transcriptomes of COVID-19 patients and found that neutrophil activation signatures were predominantly enriched in the severe COVID-19 group, which was confirmed *via* granulocyte sample validation of an independent cohort of COVID-19 patients (43). In said study, they found that alarmins S100A/8/9/12 and NETs-involved PADI4 exhibited heightened expressions of granulocytes from severe COVID-19 patients. In our study, neutrophil heterogeneity with distinct subsets of orchestrated maturation associated with KD and disease severity of COVID-19 were revealed using single-cell RNA-seq analysis in the blood of patients with these diseases. We identified a comparably heightened expression of alarmin genes S100A8/9/12 toll-like receptors TLR4/8, together with markedly increased neutrophil activation-associated signatures PADI4, ELANE, OSM, and anti-apoptotic BCL2A1, PLAC8, and CLU in neutrophil subsets from both KD and severe COVID-19 patients (**S1** cells in **Figure 6**). We also found that aged neutrophils highly expressing CXCR4 and ARG1 were concomitantly increased in PDCD1 in both KD and severe COVID-19 patients (**S5-S7** cell populations in **Figure 6**). CXCR4 phays a critical role in orchestrating the distribution and trafficking of senescent neutrophils. The over-activated/aged phenotype of neutrophils was recently shown to contribute to vascular inflammation and myocardial infarction (44) and to be associated with severity of stroke patients (45). Moreover, neutrophils with increased PDCD1 expression represented immunosuppression of CD8^+^ T cells in patients living with HIV infection (46). Our data indicated that the aged neutrophils may have an immunosuppressive effect on T cells in both KD and severe COVID-19 patients. Furthermore, MHC class II genes attributed to antigen presentation abilities were highly decreased in KD compared to FC, as well as in severe COVID-19 compared to either HC or mild COVID-19 patients (cluster 2 genes in **Figure 3**). Reduced HLA class II expression in innate myeloid cells, which is considered an established marker of immunosuppression in sepsis, may imply a dysregulated innate response to inflammation in both KD and severe COVID-19 patients. As for immunosuppressive properties, PD-L1 (CD274) is up-regulated and dysregulated in several types of immune cells of COVID-19 patients’ monocytes, neutrophils, gamma delta T cells, and CD4+ T cells. It has been demonstrated that neutrophils from severe COVID-19 patients were persistently increased in PD-L1 (CD274) expression compared to those from healthy controls by single-cell transcriptomes and proteomics (47). We confirmed those findings and further observed that elevated PD-L1 expression on neutrophils was observed in severe COVID-19 but not in KD patients. Nevertheless, the ITGA4 that was attributed to the “mildly activated” neutrophils was also reduced and clustered together with MHC class II genes in both KD and severe COVID-19 patients. FLU patients also exhibited a drastic reduction of MHC class II genes in neutrophils, but their alarmins, neutrophil activation-associated signatures, and anti-apoptotic genes were not significantly altered. Therefore, we identified that neutrophils could mediate both KD and COVID-19 immunopathology, which represents a simultaneous appearance of over activation and immunosuppressant signatures.

Collectively, we provided the first evidence using single-cell transcriptomes that KD represents similar molecular phenotypes of bidirectional over-activated neutrophils with severe COVID-19. The identification of the underlying mechanisms in immune dysfunction associated with maturation and aging of neutrophils behind KD may be essential for developing preventive strategies or focused therapies, but its etiology has remained unknown for decades. Our data implicate defective crosstalk between innate and adaptive immune responses with direct relevance for tissue destruction during the acute phase of KD. When comparing KD to severe COVID-19, the pathological features of neutrophils have important implications for diagnostic and prognostic testing. In particular, the heightened alarmins, reduced MHC class II molecules, and bidirectionally promoted neutrophil survival as well as the aging that we identified, are all crucial for clinical application for improved diagnosis and may be able to predict disease severity early in both KD and COVID-19 patients. Our study has some limitations, including the relatively low number of cases in each group and lack of a comparison with healthy children. We usually compared biochemical and molecular characteristics between Kawasaki disease with common fever to identify true features that differentiated suspected Kawasaki disease patients with febrile children for mechanistic investigation. In this study, the key innate immune response genes and abundantly expressed alarmins that currently known as important features of KD as well as those MHC class II genes were not significantly altered in febrile controls. Thus, the use of febrile children as control of KD patients is suitable at least in investing in the key molecular events occurred in KD and comparing with those in COVID-19 patients. The febrile children may have altered adaptive immune response that will take days and even a week. The altered adaptive immunity in febrile children might affected those genes that also involved in that of KD patients. A further longitudinal analysis of polymorphonuclear leukocytes of KD, febrile children and healthy controls will be required.

## Methods

### Enrollment of human subjects

We obtained ethical approval for this study from the Institutional Review Board of the Chang Gung Memorial Hospital (No. 202001350A3 and 201800472B0), as well as written informed consent from the parents or guardians of all participants. The KD patients were treated with a single dose of IVIG (2 g/kg) over a 12-hour period and aspirin. Patients whose symptoms did not fit the diagnostic criteria of KD according to the American Heart Association, had an acute fever for less than 5 days, or had an incomplete collection of pre- and post-IVIG blood samples were excluded. The patients in the fever control group had diagnoses of upper respiratory tract infection, as previously described (32).

### Preparation of single-cell suspension

Peripheral blood samples were collected from three KD patients within 24 hours before IVIG treatment and two febrile control subjects in EDTA collection tubes (vender) and centrifuged at 400 × g for 5 min at 4°C. The cell viability of purified white blood cells (WBC) exceeded 90%.

### Single-cell RNA-sequencing

Immune cell suspensions were loaded on a GemCode Single-Cell Instrument (10x Genomics, Pleasanton, CA, USA) to generate single-cell GEMs. Single-cell RNA-Seq libraries were prepared using GemCode Single-Cell 3’ Gel Bead and Library Kit (now sold as P/N 120262, 1000009, 120267, 10x Genomics). GEM-RT was performed in a Veriti 96-Well Thermal Cycler 2020/7/29 Version1 (Applied Biosystems; Model#: 9902)at 53°C for 45 min, 85°C for 5 min, and held at 4°C. After RT, GEMs were broken, and the single-strand cDNA was cleaned up with DynaBeads MyOneSilane Beads (Thermo Fisher Scientific; P/N 37002D) and the SPRIselect Reagent Kit (0.6 × SPRI; Beckman Coulter; P/N B23318). cDNA was amplified using the Veriti 96-Well Thermal Cycler Module at 98°C for 3 min, cycled 12× at 98°C for 15 s, at 67°C for 20 s, and at 72°C for 1 min and was then held at 4°C. Amplified cDNA product was cleaned up with the SPRIselect Reagent Kit (0.6×SPRI). The cDNA was subsequently sheared to ∼200 bp using a Covaris S2 Focused Ultrasonicator system (Covaris; P/N 600028). Indexed sequencing libraries were constructed using the reagents in the GemCode Single-Cell 3’ Library Kit, following these steps (1): end repair and A-tailing (2); adapter ligation (3); post-ligation cleanup with SPRIselect (4); sample index PCR and cleanup. The barcode sequencing libraries were quantified using quantitative PCR (KAPA Biosystems Library Quantification Kit for Illumina P/N KK4824 platforms). Sequencing libraries were loaded at 250 pM on an Illumina Hiseq 4000 with 2 × 150 paired-end kits using the following read lengths: 98 bp Read1, 14 bp I7 Index, 8 bp I5 Index, and 10 bp Read2.

### ScRNA-seq data analysis

We used the Cell Ranger Single Cell Pipeline v.4.0.0 to process data de-multiplexing, barcode processing, and single cell 3’ gene counting, which was generated using the 10X Chromium platform (Kawasaki disease) or downloaded from the SRA/GEO repository (COVID-19, GSE149689/PRJNA629752). All raw and processed ScRNA-seq data of KD patients and febrile controls have been deposited in the GEO database under accession number GSE200743. For all data analyses, we used publicly available software. The Loupe Cell Browser (v.5.0.1) and Seurat (v.4.0) were used for data processing, differential expression analysis, and visualization. For the scRNA-seq dataset, we removed cells with a low number of genes detected (< 200), cells with a high number of UMI detected (> 300,000), and cells with a high proportion of UMI counts attributed to mitochondrial genes (> 20%). The filtered expression matrix was then normalized and scaled to exclude unwanted sources of variation driven by the number of UMIs and mitochondrial content.

### Clustering and visualization of scRNA-seq data using t-SNE

Before clustering the cells, we ran principal component analysis (PCA) on the normalized, log-transformed, centered, and scaled gene-barcode matrix to reduce the number of feature (gene) dimensions. The pipeline adopted a python implementation of the IRLBA algorithm, which produced a projection of each cell onto the first N principal components. After running PCA, we then performed the t-distributed Stochastic Neighbor Embedding (t-SNE), a machine learning algorithm for visualization developed by Laurens van der Maaten and Geoffrey Hinton, to visualize cells in a 2-D space. Clustering was then ran in order to group cells that have similar expression profiles together based on their projection into PCA space. Two clustering methods were performed: graph-based and k-means. Cell Ranger also produced a table indicating that genes were differentially expressed in each cluster relative to all other clusters. Classification of immune cells was inferred from the annotation of cluster-specific genes and based on the expression of some well-known markers of immune cell types. Loupe™ Cell Browser (v5.0) was generally used to view the entire dataset and interactively find significant genes, cell types, and substructure within cell clusters.

### Gene Ontology enrichment analysis

Functional enrichment was performed on overlapped genesets from Gene Ontology (GO) annotation within the neutrophils by using the DAVID gene functional classification tool. The *p*-value and the Benjamini-Hochberg FDR were used to determine the significance of enrichment or the overrepresentation of terms for each annotation.

## Data availability statement

The datasets presented in this study can be found in online repositories. The names of the repository/repositories and accession number(s) can be found below: https://www.ncbi.nlm.nih.gov/geo/query/acc.cgi?acc=GSE200743.

## Ethics statement

The studies involving human participants were reviewed and approved by the Institutional Review Board of the Chang Gung Memorial Hospital. Written informed consent to participate in this study was provided by the participants’ legal guardian/next of kin.

## Author contributions

K-DC, Y-HH and H-CK contributed to the experimental design, data interpretation and writing of the manuscript. K-DC, Y-HH and W-SW contributed to statistical analysis for all genomic data and retrieved clinical data. K-DC and L-SC contributed to genomic data analysis for revision. C-LC contributed all experiments involving sample preparation and single-cell RNA sequencing. All authors contributed to the article and approved the submitted version.

## Funding

This study received funding from the following grants: MOST 108-2314-B-182 -037-MY3 from the Ministry of Science and Technology of Taiwan (MOST), and CMRPG8L0021, CMRPG8L0031, CMRPG8L0041, CMRPG8L0401, CMRPG8L0402 and CMRPG8J1641 from Chang Gung Memorial Hospital, Taiwan. Although these institutes provided financial support, they had no influence on the way in which we collected, analyzed, or interpreted the data or wrote this manuscript.

## Conflict of interest

The authors declare that the research was conducted in the absence of any commercial or financial relationships that could be construed as a potential conflict of interest.

## Publisher’s note

All claims expressed in this article are solely those of the authors and do not necessarily represent those of their affiliated organizations, or those of the publisher, the editors and the reviewers. Any product that may be evaluated in this article, or claim that may be made by its manufacturer, is not guaranteed or endorsed by the publisher.
