# Supplementary material for: Comparable bidirectional neutrophil immune dysregulation between Kawasaki disease and severe COVID-19
Source: Front Immunol. 2022 Sep 8;13:995886. doi: 10.3389/fimmu.2022.995886 (PMC9499176; doi:10.3389/fimmu.2022.995886)
Supplement: Supplementary file 1 [file DataSheet_1.docx]

**Figure S1.** **Single-cell transcriptomes of blood cells presenting different cell types.** Normalized expression of known marker genes on a tSNE plot from Kawasaki disease, febrile controls, and public available COVID-19 datasets





**Figure S2. Direct comparison of known cytokines among single-cell datasets that may be involved in hypercytokinemia of both KD and severe COVID-19 diseases.** Genes were considered differentially expressed ccording to the *P* values from the Mann-Whitney U test with a false discovery rate (FDR) ≤ 0.05.


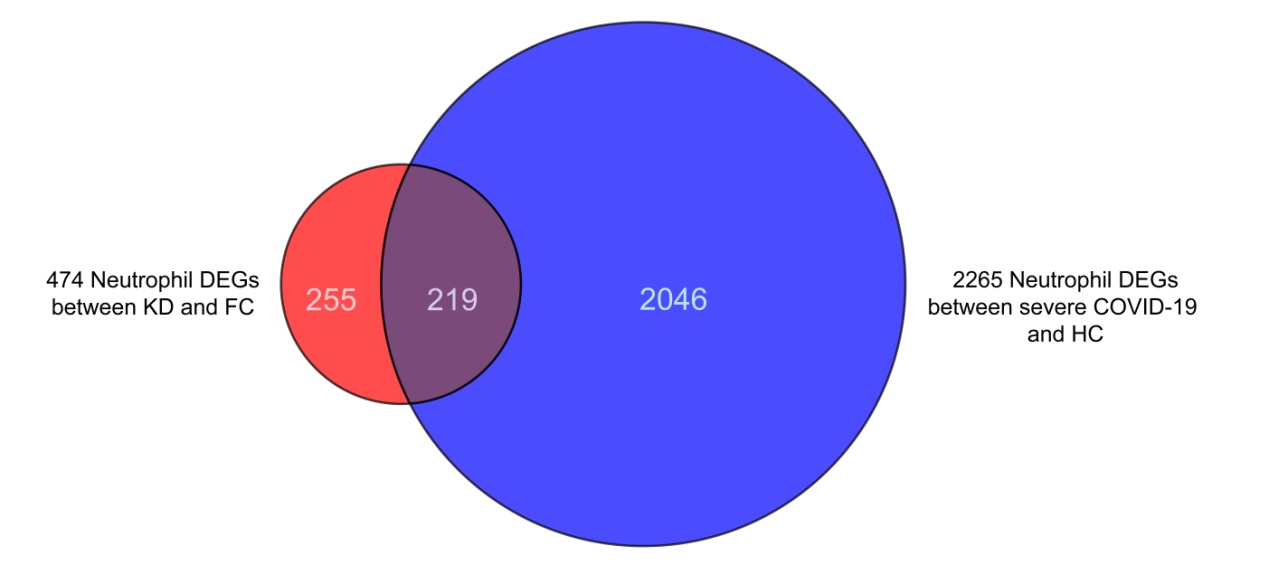


**Figure S3. Comparative transcriptome analyses of neutrophils in both KD and severe COVID-19 conditions compared to their respective control groups.** The Venn diagram illustrates an overlap of 219 neutrophil DEGs between KD and severe COVID-19 The red and blue represents neutrophil DEGs identified in KD/FC and severe COVID-19/HC transcriptome datasets. .


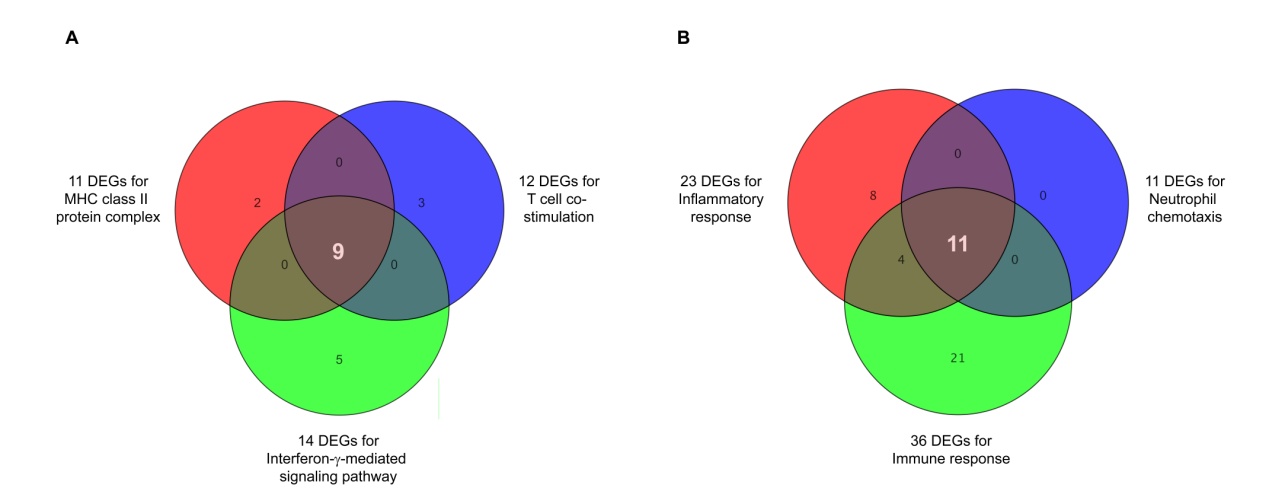


**Figure S4. Venn diagram overlap among different gene ontology terms.** **a.** Venn diagram integrating 9 specified gene expression trends from 3 gene ontology (GO) terms for antigen presentation: HLA-DMA, HLA-DMB, HLA-DPA1, HLA-DPB1, HLA-DQA1, HLA-DQB1, HLA-DRA, HLA-DRB1 and HLA-DRB5. **b.** Venn diagram integrating 11 specified gene expression trends from 3 gene ontology (GO) terms for neutrophil activation: IL1B, CXCL8, IL6, S100A8, S100A9, S100A12, CCL3L1, CCL3, CCL4, PPBP and CCL4L2.
